# Bortezomib Increased Vascular Permeability by Decreasing Cell–Cell Junction Molecules in Human Pulmonary Microvascular Endothelial Cells

**DOI:** 10.3390/ijms241310842

**Published:** 2023-06-29

**Authors:** Taichi Matsumoto, Junichi Matsumoto, Yuka Matsushita, Moeno Arimura, Kentaro Aono, Mikiko Aoki, Kazuki Terada, Masayoshi Mori, Yutaka Haramaki, Takuya Imatoh, Atsushi Yamauchi, Keisuke Migita

**Affiliations:** 1 Basic Medical Research Unit, St. Mary’s Research Center, 422, Tsubuku-honmachi, Kurume 830-8543, Fukuoka, Japan; 2 Department of Pharmaceutical Care and Health Sciences, Faculty of Pharmaceutical Sciences, Fukuoka University, 8-19-1, Nanakuma, Jonan-ku, Fukuoka 814-0180, Fukuoka, Japan; jmatsumoto@fukuoka-u.ac.jp (J.M.); blue230688k@icloud.com (K.A.); atyama@fukuoka-u.ac.jp (A.Y.); 3 Department of Drug Informatics and Translational Research, Faculty of Pharmaceutical Sciences, Fukuoka University, 8-19-1, Nanakuma, Jonan-ku, Fukuoka 814-0180, Fukuoka, Japanimatoh@fukuoka-u.ac.jp (T.I.); migitak@fukuoka-u.ac.jp (K.M.); 4 Department of Pathology, Faculty of Medicine, Fukuoka University, 7-45-1, Nanakuma, Jonan-ku, Fukuoka 814-0180, Fukuoka, Japan; mikikoss@fukuoka-u.ac.jp; 5 Division of Pharmacotherapeutics, Faculty of Pharmaceutical Sciences, Himeji Dokkyo University, 7-2-1, Kamiohno, Himeji 670-8524, Hyogo, Japan; kterada@gm.himeji-du.ac.jp; 6 Department of Pharmacotherapeutics, Faculty of Pharmaceutical Sciences, Fukuoka University, 8-19-1, Nanakuma, Jonan-ku, Fukuoka 814-0180, Fukuoka, Japan; morimasa@fukuoka-u.ac.jp; 7 Psychology Program, Graduate School of Humanities and Social Sciences, Hiroshima University, Kagamiyama, 1-1-1, Kagamiyama, Higashi-Hiroshima City 739-8512, Hiroshima, Japan; haramaki@hiroshima-u.ac.jp

**Keywords:** bortezomib, vascular permeability, adhesion molecule

## Abstract

Bortezomib (BTZ), a chemotherapeutic drug used to treat multiple myeloma, induces life-threatening side effects, including severe pulmonary toxicity. However, the mechanisms underlying these effects remain unclear. The objectives of this study were to (1) investigate whether BTZ influences vascular permeability and (2) clarify the effect of BTZ on the expression of molecules associated with cell–cell junctions using human pulmonary microvascular endothelial cells in vitro. Clinically relevant concentrations of BTZ induced limited cytotoxicity and increased the permeability of human pulmonary microvascular endothelial cell monolayers. BTZ decreased the protein expression of claudin-5, occludin, and VE-cadherin but not that of ZO-1 and β-catenin. Additionally, BTZ decreased the mRNA expression of claudin-5, occludin, ZO-1, VE-cadherin, and β-catenin. Our results suggest that BTZ increases the vascular permeability of the pulmonary microvascular endothelium by downregulating cell–cell junction molecules, particularly claudin-5, occludin, and VE-cadherin.

## 1. Introduction

Bortezomib (BTZ) was approved by the United States Food and Drug Administration in 2003 as a breakthrough treatment for multiple myeloma (MM) [1]. BTZ inhibits the activity of the proteasome, which subsequently inhibits the signaling pathway of the transcription factor nuclear factor-kappa B (NF-κB) [2,3]. NF-κB has anti-apoptotic effects [4]; thus, BTZ induces apoptosis and can sensitize cells to other cancer treatments [3]. BTZ is widely used to treat MM but can induce various adverse events that potentially lead to early discontinuation of therapy, which negatively affects the quality of life and outcomes of patients [5]. Among BTZ-related adverse effects, pulmonary toxicity is rare but life-threatening [6,7,8]. Although steroidal anti-inflammatory drugs are used to treat BTZ-induced pulmonary toxicity, a proportion of patients are resistant to steroid therapy [7,9,10]. Additionally, specific methods are not available to treat or prevent this BTZ-induced severe pulmonary toxicity because the mechanism is unclear.

Mukai et al. reported that pulmonary toxicity accompanying increased vascular permeability is a characteristic side effect of BTZ [11]. We previously demonstrated that BTZ enhances hematopoietic stem cell mobilization by increasing vascular permeability in the bone marrow [12]. Based on these results, we conceived that BTZ-induced pulmonary toxicity is caused by increased vascular permeability of the pulmonary endothelium. 

In the present study, to address the mechanism of BTZ-induced pulmonary toxicity, we investigated whether BTZ increases vascular permeability and its underlying mechanism through in vitro experiments using human pulmonary microvascular endothelial cells (HPMECs).

## 2. Results

### 2.1. Cytotoxicity of BTZ towards HPMEC Monolayers

**Figure 1:**
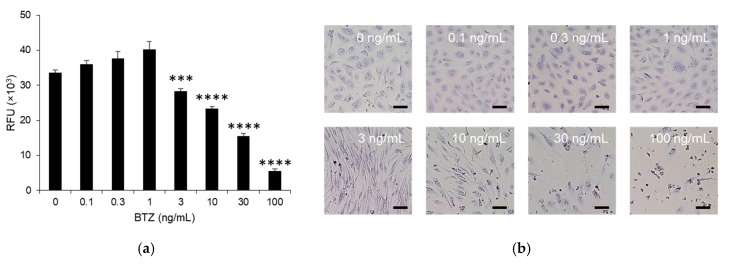
Effect of BTZ on the viability of the HPMEC monolayer. (**a**) HPMEC monolayer treated with 0–10 ng/mL BTZ for 72 h. Viability was determined in a PrestoBlue cell viability assay, and the fluorescent intensity was measured. Experiments were repeated three times, and data are expressed as the mean ± SD. Significant differences were analyzed using one-way analysis of variance, followed by Dunnett’s multiple comparisons test. **** *p* < 0.001, *** *p* < 0.005. RFU, relative fluorescence intensity. (**b**) Morphology of the HPMEC monolayer treated with 0–10 ng/mL for 72 h. Magnification: 20×; scale bar: 50 μm.

BTZ is normally administered intravenously or subcutaneously at 1.3 mg/m^2^. In both cases, the plasma concentration of BTZ is maintained at approximately 1 ng/mL during the elimination phase until at least 72 h after administration [13,14,15]. Therefore, we treated HPMEC monolayers with 1–10 ng/mL BTZ for 72 h. No cytotoxicity was observed in the HPMEC monolayer at concentrations of ≤1 ng/mL BTZ (Figure 1a). Treatment with 3 ng/mL BTZ led to a slight but significant reduction in the viability of HPMECs (Figure 1a). In contrast, ≥10 ng/mL BTZ strongly reduced the viability of HPMEC monolayers in a dose-dependent manner (Figure 1a). Differences were not observed in the morphology of the HPMEC monolayer treated with ≤1 ng/mL BTZ compared to that treated with the vehicle (Figure 1b). Although significant differences likely did not occur in the number of HPMECs treated with the vehicle or 3 ng/mL BTZ, HPMECs became spindle-shaped with the administration of ≥3 ng/mL BTZ (Figure 1b). The number of HPMECs decreased after treatment with 10 ng/mL BTZ (Figure 1b). Therefore, we treated the HPMEC monolayer with ≤3 ng/mL BTZ in subsequent experiments.

### 2.2. Effect of BTZ on the Permeability of HPMEC Monolayers

**Figure 2:**
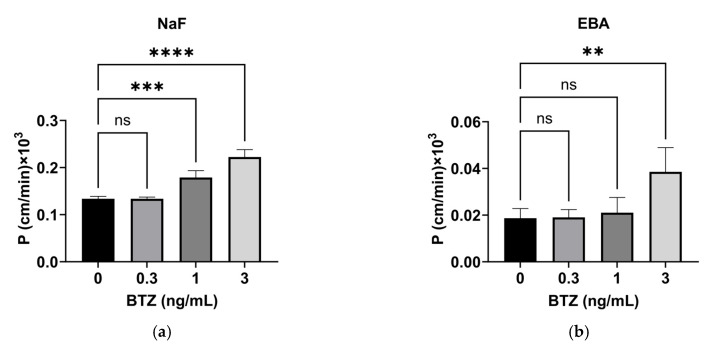
Effect of BTZ on the permeability of HPMEC monolayers. (**a**,**b**) Permeability of NaF (**a**) and EBA (**b**) through HPMEC monolayers treated with the indicated concentrations of BTZ for 72 h. P, permeability efficiency. The calculation method has been described previously [17]. Both experiments were conducted three times, and data are expressed as the mean ± SD. Significant differences were analyzed via one-way analysis of variance followed by Dunnett’s multiple comparisons test. **** *p* < 0.001, *** *p* < 0.005, ** *p* < 0.01.; ns = not significant.

The effect of BTZ on the permeability of HPMEC monolayers was investigated using a permeability assay with two different-sized substrates: NaF- and Evans blue-conjugated bovine serum albumin (EBA) have molecular weights of 376 and 67,000 Da and were used as small- and large-molecule models, respectively [16]. NaF permeability was not affected by 0.3 ng/mL BTZ but increased with ≥1 ng/mL BTZ, indicating that this effect of BTZ was dose-dependent (Figure 2a). In addition, EBA permeability was not affected by ≤1 ng/mL BTZ but was significantly increased by 3 ng/mL BTZ (Figure 2b). These results indicate that BTZ doses relevant to the clinical setting increased the permeability of both small and large molecules while causing minimal cytotoxicity.

### 2.3. Effect of BTZ on Adhesion Molecule Expression in HPMECs

**Figure 3:**
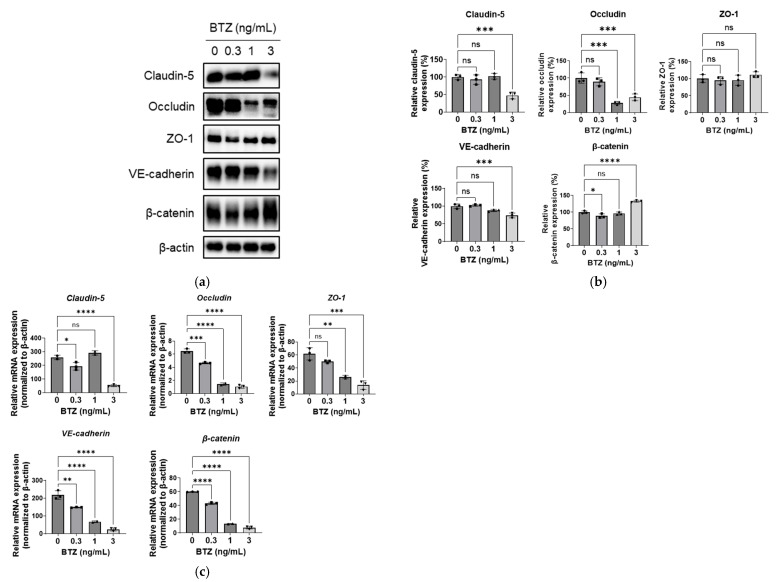
Effect of BTZ on the expression of adhesion molecules in HPMECs (**a**–**c**) HPMECs were treated with BTZ at the indicated concentrations for 72 h, and the protein (**a**,**b**) and mRNA (**c**) expression levels of claudin-5, occludin, ZO-1, VE-cadherin, and β-catenin were analyzed. (**a**) Representative blot for Western blot analysis. β-Actin was detected as a reference. (**b**) Relative expression of the five proteins normalized to the expression level of β-actin. (**c**) mRNA expression level of the five adhesion molecule genes normalized to that of the β-actin gene. Both protein and mRNA expression experiments were conducted three times, and data are expressed as the mean ± SD. Significant differences were analyzed via one-way analysis of variance, followed by Dunnett’s multiple comparisons test. **** *p* < 0.001, *** *p* < 0.005, ** *p* < 0.01, and * *p* < 0.05; ns = not significant.

Various adhesion molecules are associated with the regulation of lung endothelium permeability [18]. We investigated the effects of BTZ on the expression of the adhesion molecules related to tight junctions (claudin-5, occludin, and ZO-1) and adherence junctions (VE-cadherin and β-catenin) in HPMECs. BTZ at 0.3 ng/mL had no effects on the expression of these five molecules (Figure 3a,b); BTZ at 1 ng/mL significantly reduced occludin expression (Figure 3a,b); and BTZ at 3 ng/mL significantly reduced claudin-5, occludin, and VE-cadherin expression but increased β-catenin expression in HPMECs (Figure 3a,b). BTZ did not affect ZO-1 expression at any of the tested doses. 

Next, we investigated whether BTZ affected the mRNA expression of adhesion molecules. BTZ at 0.3 ng/mL reduced the mRNA expression of occludin, VE-cadherin, and β-catenin (Figure 3c); BTZ at 1 ng/mL reduced the mRNA expression of occludin, ZO-1, VE-cadherin, and β-catenin (Figure 3c); and BTZ at 3 ng/mL reduced the expression of all five genes (Figure 3c). 

## 3. Discussion

BTZ induces severe pulmonary toxicity in patients with MM; however, the underlying mechanism is unclear. Increased vascular permeability is a characteristic feature of BTZ-induced pulmonary toxicity. We previously demonstrated that BTZ enhances hematopoietic stem cell mobilization by increasing vascular permeability in the bone marrow. This study was performed to confirm whether BTZ increases vascular permeability. The in vitro permeability assay showed that BTZ at clinically relevant concentrations increased the permeability of small and large molecules through the HPMEC monolayer in a dose-dependent manner with low cytotoxicity (Figure 1 and Figure 2). A previous study reported that BTZ increased the vascular permeability of the model large-molecule dextran through a human umbilical vein endothelial cell (HUVEC) monolayer [19]; however, the BTZ concentration tested in the study was lethal towards HUVECs, suggesting that the increased permeability was caused by cell death. In contrast, we found that BTZ increased the permeability of clinically relevant concentrations of both small and large molecules through an HPMEC monolayer with minimal cytotoxicity. To our knowledge, this is the first study to demonstrate that a clinically valid dose of BTZ can increase vascular permeability. Our previous study demonstrated via an animal study that BTZ increases the permeability of Evans blue dye, a model for small molecules, in the bone marrow [12]. In our ongoing in vivo study, we are investigating whether BTZ increases the permeability of large molecules in the bone marrow and pulmonary microvascular endothelium.

We also examined the mechanism underlying the ability of BTZ to increase vascular permeability in HPMECs. Our results show that BTZ significantly decreased the expression of claudin-5, occludin, and VE-cadherin (Figure 3). Treatment of the HPMEC monolayer with 1 ng/mL BTZ increased the permeability of NaF but not EBA (Figure 2a) and downregulated occludin but not the other molecules (Figure 3b). In contrast, treatment of the HPMEC monolayer with 3 ng/mL BTZ increased the permeability of NaF and EBA (Figure 2b) and downregulated occludin, claudin-5, and VE-cadherin (Figure 3b). These results suggest that BTZ increases the permeability of small molecules by downregulating occludin and increases the permeability of large molecules by downregulating claudin-5 and VE-cadherin. The plasma concentration of BTZ is normally maintained at approximately 1 ng/mL during the elimination phase at the typical dose of BTZ of 1.3 mg/m^2^ in patients with MM [13,14,15]. However, reports have shown that the plasma concentration of BTZ was increased by concomitant administration of the CYP3A4 inhibitor ketoconazole because BTZ is a substrate of CYP3A4 [17]. Previous studies have not investigated the influence of ketoconazole on the pathogenesis of BTZ-induced pulmonary toxicity. BTZ has been identified as a substrate of CYP2C19, CYP1A2, CYP2D6, and CYP2C9 [20,21]. Clinical studies are required to investigate the relationship of BTZ pharmacokinetics and BTZ-induced pulmonary toxicity in patients with MM.

An interesting finding in this study was the decrease in claudin-5 by BTZ because claudin-5 has recently been identified as a target for improving drug delivery to the brain. The blood–brain barrier (BBB) is a highly selective semipermeable layer of microvascular endothelial cells that regulates the passage of solutes from the blood to the brain [22]. Therefore, the modulation of BBB permeability is critical for developing drugs that target the central nervous system. Claudin-5 is prominently expressed in brain microvascular endothelial cells but not in brain parenchyma or choroid plexus epithelial cells, making it an ideal therapeutic target for the modulation of BBB permeability [23,24]. A recent study demonstrated that an anti-claudin-5 antibody increased BBB permeability with no behavioral changes or changes in plasma biomarkers of inflammation, liver, or kidney injury, suggesting that claudin-5 can be targeted to enhance drug delivery to the brain [25,26]. Because BTZ is a cytotoxic anticancer agent, it cannot be administered as a BBB permeability-enhancing agent in patients without cancer. Determining the mechanism by which BTZ decreases the expression of claudin-5 but also occludin and VE-cadherin may lead to the development of drugs that enhance drug delivery to the brain.

The mRNA expression of five junction molecules was dose-dependently decreased in BTZ-treated HPMECs (Figure 3c). Because BTZ induces the accumulation of nuclear factor of kappa light polypeptide gene enhancer in B-cell inhibitor alpha (IκBα) via proteasome inhibition, BTZ abrogates NF-κB signaling. Our database analysis showed that the promoter regions of the five genes contained a binding site for NF-κB (Figure S1). Therefore, BTZ-induced NF-κB inhibition may be associated with the decreased mRNA expression of the five molecules. However, previous studies reported that NF-κB activation inhibited the expression of claudin-5 [27,28], occludin [28], ZO-1 [29], and VE-cadherin [30]. The pathway by which BTZ downregulates the expression of cell–cell adhesion molecules should be further investigated. 

We also observed that BTZ increased the expression of β-catenin protein in a dose-dependent manner (Figure 3a,b), which may have been caused by BTZ-induced inhibition of proteasomes because β-catenin is a target of the ubiquitin–proteasome pathway [31]. Similar to our findings, a previous study demonstrated that BTZ accumulated β-catenin in human mesenchymal stem cells and osteoblast-like cells [32]. β-catenin promotes adherence junction formation by binding to VE-cadherin [33]. BTZ increased β-catenin expression but decreased VE-cadherin expression in HPMECs, and these changes could be involved in the increased permeability of the HPMEC barrier.

The present study found that BTZ increased the permeability of small and large molecules through HPMEC monolayers and decreased cell–cell junction molecules; however, the association of increased permeability with decreased expression of adhesion molecules is unclear. Further studies are required to clarify the mechanism by which BTZ decreases the expression of adhesion molecules. Such studies could lead to the development of agents that prevent or treat BTZ-induced pulmonary toxicity in patients with MM. In addition, whether BTZ increases the permeability of pulmonary microvasculature under physiological conditions must be further investigated. Three-dimensional porous scaffold technology has the ability to mimic the pulmonary vasculature and could represent a useful tool to investigate the effect of BTZ on vascular permeability under physiological conditions [34]. Furthermore, computational simulations/in silico studies combined with three-dimensional porous scaffold technology [35,36,37,38,39] could provide a better understanding of the mechanism by which BTZ increases vascular permeability.

Collectively, our results demonstrate that BTZ increased vascular permeability in the HPMEC barrier at concentrations that had little effect on cell viability and suggest that BTZ-induced increased permeability was caused by a decrease in claudin-5, occludin, and VE-cadherin. These findings provide a basis for developing agents for preventing and treating BTZ-induced pulmonary toxicity.

## 4. Materials and Methods

### 4.1. Culture of HPMECs

HPMECs were purchased from Takara Bio, Inc. (Shiga, Japan). HPMECs were cultured in Endothelial Cell Growth Medium MV (EGM MV) Kit (Takara Bio, Inc.) at 37 °C in a humid atmosphere with 5% CO_2_. Culture dishes and plates were coated with Cellmatrix Type I-C, a collagen type I product, at 0.3 mg/mL in 1 N HCl. HPMECs from passages lower than passage five were used in all experiments.

### 4.2. Viability Assay

HPMECs (1.9 × 10^4^ cells) resuspended in 100 μL of EGM MV were seeded on collagen I-coated 96-well plates and cultured for 3 days to form a tight monolayer. The culture medium was replaced with 100 μL of fresh EGM MV containing various concentrations of BTZ. Three days later, cell viability was determined using the PrestoBlue Cell Viability Reagent (Thermo Fisher Scientific K.K., Osaka, Japan).

### 4.3. Giemsa Staining

The cells were washed with PBS and fixed with methanol for 5 min at 24 °C. The cells were dried for 10 min at 24 °C and immersed in 20-fold diluted Giemsa Staining Solution (MUTO PURE CHEMICALS Co., Ltd., Tokyo, Japan) for 20 min at 24 °C. The cells were washed with tap water and rinsed with double-distilled water. After drying, images of the cells were captured at a magnification of 20× using a BZ-X810 microscope (KEYENCE CORPORATION, Osaka, Japan). Representative images are shown. 

### 4.4. Permeability Assay

HPMECs (1.9 × 10^4^ cells) resuspended in 100 μL of EGM MV were seeded onto a collagen I-coated Transwell insert with 0.4 μm pores (Corning, Inc., Corning, NY, USA). The Transwell inserts were placed in a 24-well plate containing 600 μL of EGM MV and cultured for 3 days to form a tight monolayer. The media on the Transwell inserts were replaced with fresh EGM MV containing NaF, EBA, and various concentrations of BTZ. The amounts of NaF and EBA in the lower chamber that passed through the HPMEC monolayer were quantified by measuring the fluorescence intensity. Using the obtained data, permeability clearance was calculated as described previously [17].

### 4.5. Western Blotting

After treating HPMECs with 0, 0.3, 1, and 3 ng/mL BTZ for 72 h, cells were washed with ice-cold phosphate-buffered saline and solubilized in radioimmunoprecipitation buffer (25 mM Tris-HCl, 150 mM NaCl, 1% NP-40, 1% sodium deoxycholate, and 0.1% sodium dodecyl sulfate) supplemented with complete mini (Sigma-Aldrich, St. Louis, MO, USA) and phosSTOP (Sigma-Aldrich). After sonication, the cell lysates were centrifuged at 14,000× *g* for 20 min at 4 °C, and the supernatants were used for analysis. The protein concentrations of the lysates were measured using a Pierce™ BCA Protein Assay Kit (Thermo Fisher Scientific). The protein sample (10 μg) was treated with the Blue Loading Buffer Pack (Cell Signaling Technology, Danvers, MA, USA) for 5 min at 95 °C and subjected to sodium dodecyl sulfate–polyacrylamide gel electrophoresis, with a gel run for 1 h at 200 V. The proteins were transferred to a polyvinylidene fluoride (PVDF Transfer Membrane, 0.2 µm (Bio-Rad Laboratories, Hercules, CA, USA) using a Transblot SD cell (Bio-Rad Laboratories) for 1 h at 10 V. After washing with Tris-buffered saline containing 0.05% Tween 20 (TBST) for 5 min with agitation three times, the membrane surface was blocked with TBST supplemented with 5% skim milk (FUJIFILM Wako Pure Chemical Corporation, Osaka, Japan) for 1 h at 24 °C, and then immersed in TBST + 1% skim milk supplemented with primary antibody overnight at 4 °C with agitation. The following primary antibodies were used: claudin 5 monoclonal antibody (4C3C2) (Thermo Fisher Scientific, Cat#:35-2500), occludin (E6B4R) rabbit mAb (Cell Signaling Technology, Cat#:91131), ZO-1 (D7D12) rabbit mAb (Cell Signaling Technology, Cat#:8193), VE-cadherin (D87F2) XP^®^ rabbit mAb (Cell Signaling Technology, Cat#:2500), β-catenin (D10A8) XP^®^ rabbit mAb (Cell Signaling Technology, Cat#:8480), and anti-beta actin antibody (Abcam plc., Cambridge, UK, Cat#:8227). After washing it three times with TBST for 5 min with agitation, the membrane was immersed in TBST + 1% skim milk supplemented with a secondary antibody for 1 h at 24 °C. The secondary antibodies used were anti-rabbit IgG and horseradish-peroxidase-linked anti-rabbit IgG (Cell Signaling Technology, Cat#7074), or anti-mouse IgG (Cell Signaling Technology, Cat#7076). After washing it with TBST for 5 min with agitation three times, the membrane was immersed in electrochemiluminescence reagent composed of 1 M Tris-HCl (pH 8.8) supplemented with 1.25 mM luminol (Tokyo Chemical Industry Co., Ltd., Tokyo, Japan), 0.2 mM p-coumaric acid (Sigma-Aldrich), and 0.004% H_2_O_2_ (FUJIFILM Wako Pure Chemical Corporation). Chemiluminescence was visualized using a ChemiDoc Touch Imaging System (Bio-Rad Laboratories). The density of the blot bands was quantified using ImageJ software (https://imagej.nih.gov/ij/download.html (accessed on 27 June 2023)).

### 4.6. Quantitative Real-Time Polymerase Chain Reaction

After treating HPMECs with 0, 0.3, 1, and 3 ng/mL BTZ for 72 h, total RNA was extracted using a NucleoSpin RNA Kit (Takara Bio, Inc.). cDNA was synthesized from total RNA using ReverTra Ace qPCR RT Master Mix with gDNA Remover (TOYOBO Co., Ltd., Osaka, Japan). cDNA amplification by THUNDERBIRD^®^ SYBR qPCR Mix (TOYOBO Co., Ltd.) and monitoring of SYBR fluorescence were performed using a StepOne Plus Real-Time PCR System (Thermo Fisher Scientific).

### 4.7. Statistical Analysis

Statistical significance was determined using one-way analysis of variance followed by Dunnett’s post hoc test using GraphPad Prism 9.5.0 software (GraphPad, Inc., La Jolla, CA, USA). Statistical significance was set at *p* < 0.05.

## 5. Conclusions

BTZ increases the permeability of small and large molecules through the HPMEC barrier by suppressing cell–cell adhesion molecules, such as occludin, clausin-5, and VE-cadherin.

## Data Availability

DNA sequences 250 bases upstream and downstream of the genes were obtained from the DBTSS (http://dbtss.hgc.jp/ (accessed on 27 June 2023)). DNA sequences containing the binding sites for p50 and p60 were analyzed using JASPAR 2016 (http://jaspar2016.genereg.net/ (accessed on 27 June 2023)).

## Supplementary Materials

The supporting information can be downloaded from https://www.mdpi.com/article/10.3390/ijms241310842/s1.

## References

[1] Kane R.C., Bross P.F., Farrell A.T., Pazdur R. (2003). Velcade: U.S. FDA approval for the treatment of multiple myeloma progressing on prior therapy. Oncologist.

[2] Barnes P.J., Karin M. (1997). Nuclear factor-kappaB: A pivotal transcription factor in chronic inflammatory diseases. N. Engl. J. Med..

[3] Adams J. (2001). Proteasome inhibition in cancer: Development of PS-341. Semin. Oncol..

[4] Hideshima T., Richardson P., Chauhan D., Palombella V.J., Elliott P.J., Adams J., Anderson K.C. (2001). The proteasome inhibitor PS-341 inhibits growth, induces apoptosis, and overcomes drug resistance in human multiple myeloma cells. Cancer Res..

[5] Pancheri E., Guglielmi V., Wilczynski G.M., Malatesta M., Tonin P., Tomelleri G., Nowis D., Vattemi G. (2020). Non-Hematologic Toxicity of Bortezomib in Multiple Myeloma: The Neuromuscular and Cardiovascular Adverse Effects. Cancers.

[6] Boyer J.E., Batra R.B., Ascensao J.L., Schechter G.P. (2006). Severe pulmonary complication after bortezomib treatment for multiple myeloma. Blood.

[7] Miyakoshi S., Kami M., Yuji K., Matsumura T., Takatoku M., Sasaki M., Narimatsu H., Fujii T., Kawabata M., Taniguchi S. (2006). Severe pulmonary complications in Japanese patients after bortezomib treatment for refractory multiple myeloma. Blood.

[8] Chew E., Filshie R., Wei A. (2007). Development of fatal bortezomib induced acute lung injury despite concurrent therapy with high-dose dexamethasone. Leuk. Lymphoma.

[9] Kharel P., Uprety D., Chandra A.B., Hu Y., Belur A.A., Dhakal A. (2018). Bortezomib-Induced Pulmonary Toxicity: A Case Report and Review of Literature. Case Rep. Med..

[10] Duek A., Feldberg E., Haran M., Berrebi A. (2007). Pulmonary fibrosis in a myeloma patient on bortezomib treatment. A new severe adverse effect of a new drug. Am. J. Hematol..

[11] Mukai H., Ohyashiki K., Katoh T., Kusumoto M., Gemma A., Sakai H., Sugiyama Y., Hatake K., Fukuda Y., Kudoh S. (2011). Lung injury associated with bortezomib therapy in Japan. Rinsho Ketsueki.

[12] Matsumoto T., Takamatsu Y., Moriyama H., Terada K., Mori M., Ono K., Migita K., Hara S. (2021). Bortezomib enhances G-CSF-induced hematopoietic stem cell mobilization by decreasing CXCL12 levels and increasing vascular permeability. Exp. Hematol..

[13] Papandreou C.N., Daliani D.D., Nix D., Yang H., Madden T., Wang X., Pien C.S., Millikan R.E., Tu S.M., Pagliaro L. (2004). Phase I trial of the proteasome inhibitor bortezomib in patients with advanced solid tumors with observations in androgen-independent prostate cancer. J. Clin. Oncol..

[14] Moreau P., Coiteux V., Hulin C., Leleu X., van de Velde H., Acharya M., Harousseau J.L. (2008). Prospective comparison of subcutaneous versus intravenous administration of bortezomib in patients with multiple myeloma. Haematologica.

[15] Leal T.B., Remick S.C., Takimoto C.H., Ramanathan R.K., Davies A., Egorin M.J., Hamilton A., LoRusso P.A., Shibata S., Lenz H.J. (2011). Dose-escalating and pharmacological study of bortezomib in adult cancer patients with impaired renal function: A National Cancer Institute Organ Dysfunction Working Group Study. Cancer Chemother. Pharmacol..

[16] Takata F., Dohgu S., Yamauchi A., Matsumoto J., Machida T., Fujishita K., Shibata K., Shinozaki Y., Sato K., Kataoka Y. (2013). In vitro blood-brain barrier models using brain capillary endothelial cells isolated from neonatal and adult rats retain age-related barrier properties. PLoS ONE.

[17] Venkatakrishnan K., Rader M., Ramanathan R.K., Ramalingam S., Chen E., Riordan W., Trepicchio W., Cooper M., Karol M., von Moltke L. (2009). Effect of the CYP3A inhibitor ketoconazole on the pharmacokinetics and pharmacodynamics of bortezomib in patients with advanced solid tumors: A prospective, multicenter, open-label, randomized, two-way crossover drug-drug interaction study. Clin. Ther..

[18] Shasby D.M. (2007). Cell-cell adhesion in lung endothelium. Am. J. Physiol. Lung Cell. Mol. Physiol..

[19] Tamura D., Arao T., Tanaka K., Kaneda H., Matsumoto K., Kudo K., Aomatsu K., Fujita Y., Watanabe T., Saijo N. (2010). Bortezomib potentially inhibits cellular growth of vascular endothelial cells through suppression of G2/M transition. Cancer Sci..

[20] Uttamsingh V., Lu C., Miwa G., Gan L.S. (2005). Relative contributions of the five major human cytochromes P450, 1A2, 2C9, 2C19, 2D6, and 3A4, to the hepatic metabolism of the proteasome inhibitor bortezomib. Drug Metab. Dispos..

[21] Fatunde O.A., Brown S.A. (2020). The Role of CYP450 Drug Metabolism in Precision Cardio-Oncology. Int. J. Mol. Sci..

[22] Banks W.A. (2016). From blood-brain barrier to blood-brain interface: New opportunities for CNS drug delivery. Nat. Rev. Drug Discov..

[23] Vanlandewijck M., He L., Mae M.A., Andrae J., Ando K., Del Gaudio F., Nahar K., Lebouvier T., Lavina B., Gouveia L. (2018). A molecular atlas of cell types and zonation in the brain vasculature. Nature.

[24] Castro Dias M., Coisne C., Lazarevic I., Baden P., Hata M., Iwamoto N., Francisco D.M.F., Vanlandewijck M., He L., Baier F.A. (2019). Claudin-3-deficient C57BL/6J mice display intact brain barriers. Sci. Rep..

[25] Tachibana K., Hashimoto Y., Shirakura K., Okada Y., Hirayama R., Iwashita Y., Nishino I., Ago Y., Takeda H., Kuniyasu H. (2021). Safety and efficacy of an anti-claudin-5 monoclonal antibody to increase blood-brain barrier permeability for drug delivery to the brain in a non-human primate. J. Control. Release.

[26] Hashimoto Y., Zhou W., Hamauchi K., Shirakura K., Doi T., Yagi K., Sawasaki T., Okada Y., Kondoh M., Takeda H. (2018). Engineered membrane protein antigens successfully induce antibodies against extracellular regions of claudin-5. Sci. Rep..

[27] Clark P.R., Kim R.K., Pober J.S., Kluger M.S. (2015). Tumor necrosis factor disrupts claudin-5 endothelial tight junction barriers in two distinct NF-kappaB-dependent phases. PLoS ONE.

[28] Ding Y.X., Eerduna G.W., Duan S.J., Li T., Liu R.X., Zhang L.M., Wang T., Fu F.H. (2021). Escin ameliorates the impairments of neurological function and blood brain barrier by inhibiting systemic inflammation in intracerebral hemorrhagic mice. Exp. Neurol..

[29] Ibrahim S., Zhu X., Luo X., Feng Y., Wang J. (2020). PIK3R3 regulates ZO-1 expression through the NF-kB pathway in inflammatory bowel disease. Int. Immunopharmacol..

[30] Colas-Algora N., Garcia-Weber D., Cacho-Navas C., Barroso S., Caballero A., Ribas C., Correas I., Millan J. (2020). Compensatory increase of VE-cadherin expression through ETS1 regulates endothelial barrier function in response to TNFalpha. Cell. Mol. Life Sci..

[31] Aberle H., Bauer A., Stappert J., Kispert A., Kemler R. (1997). beta-catenin is a target for the ubiquitin-proteasome pathway. EMBO J..

[32] Qiang Y.W., Hu B., Chen Y., Zhong Y., Shi B., Barlogie B., Shaughnessy J.D. (2009). Bortezomib induces osteoblast differentiation via Wnt-independent activation of beta-catenin/TCF signaling. Blood.

[33] Guo M., Breslin J.W., Wu M.H., Gottardi C.J., Yuan S.Y. (2008). VE-cadherin and beta-catenin binding dynamics during histamine-induced endothelial hyperpermeability. Am. J. Physiol. Cell. Physiol..

[34] Prakoso A.T., Basri H., Adanta D., Yani I., Ammarullah M.I., Akbar I., Ghazali F.A., Syahrom A., Kamarul T. (2023). The Effect of Tortuosity on Permeability of Porous Scaffold. Biomedicines.

[35] Ammarullah M.I., Afif I.Y., Maula M.I., Winarni T.I., Tauviqirrahman M., Akbar I., Basri H., van der Heide E., Jamari J. (2021). Tresca Stress Simulation of Metal-on-Metal Total Hip Arthroplasty during Normal Walking Activity. Materials.

[36] Ammarullah M.I., Hartono R., Supriyono T., Santoso G., Sugiharto S., Permana M.S. (2023). Polycrystalline Diamond as a Potential Material for the Hard-on-Hard Bearing of Total Hip Prosthesis: Von Mises Stress Analysis. Biomedicines.

[37] Ammarullah M.I., Santoso G., Sugiharto S., Supriyono T., Wibowo D.B., Kurdi O., Tauviqirrahman M., Jamari J. (2022). Minimizing Risk of Failure from Ceramic-on-Ceramic Total Hip Prosthesis by Selecting Ceramic Materials Based on Tresca Stress. Sustainability.

[38] Ammarullah M.I., Santoso G., Sugiharto S., Supriyono T., Kurdi O., Tauviqirrahman M., Winarni T.I., Jamari J. (2022). Tresca stress study of CoCrMo-on-CoCrMo bearings based on body mass index using 2D computational model. J. Tribol..

[39] Salaha Z.F.M., Ammarullah M.I., Abdullah N., Aziz A.U.A., Gan H.S., Abdullah A.H., Abdul Kadir M.R., Ramlee M.H. (2023). Biomechanical Effects of the Porous Structure of Gyroid and Voronoi Hip Implants: A Finite Element Analysis Using an Experimentally Validated Model. Materials.

