# Supplementary material for: Bortezomib Increased Vascular Permeability by Decreasing Cell–Cell Junction Molecules in Human Pulmonary Microvascular Endothelial Cells
Source: Int J Mol Sci. 2023 Jun 29;24(13):10842. doi: 10.3390/ijms241310842 (PMC10342080; doi:10.3390/ijms241310842)
Supplement: Supplementary file 1 [file ijms-24-10842-s001.zip › ijms-2469414-supplementary.pdf]

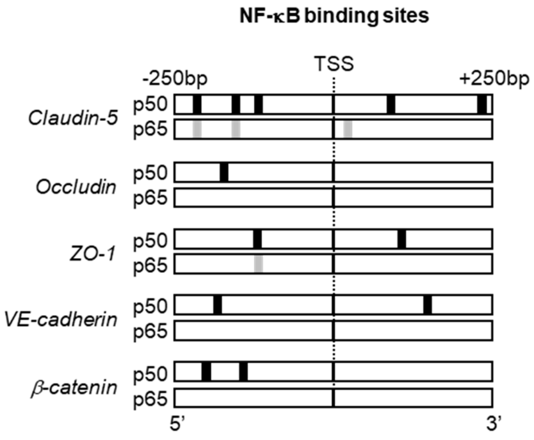

Figure S1. Binding sites for NF-κB in five adhesion molecule genes. We sought to identify the binding sequence for p50 (black regions) and p65 (gray regions), which are components of NF-κB, among 250 bases upstream and downstream of the putative transcription start site (TSS) of the claudin-5, occludin, ZO-1, VE-cadherin, and β-catenin genes.
